# The type of Aβ-related neuronal degeneration differs between amyloid precursor protein (APP23) and amyloid β-peptide (APP48) transgenic mice

**DOI:** 10.1186/2051-5960-1-77

**Published:** 2013-11-18

**Authors:** Ajeet Rijal Upadhaya, Frederik Scheibe, Irina Kosterin, Dorothee Abramowski, Janina Gerth, Sathish Kumar, Stefan Liebau, Haruyasu Yamaguchi, Jochen Walter, Matthias Staufenbiel, Dietmar Rudolf Thal

**Affiliations:** Institute of Pathology - Laboratory of Neuropathology, University of Ulm, Ulm, Germany; Novartis Institutes for Biomedical Research, Basel, Switzerland; Department of Neurology, University of Bonn, Bonn, Germany; Institute of Anatomy and Cell Biology, University of Ulm, Ulm, Germany; Gunma University School of Health Sciences, Gunma, Japan

**Keywords:** Intracellular amyloid β-protein, Extracellular amyloid β-protein, Mitochondria, Dendrites, Toxicity, Degeneration

## Abstract

**Background:**

The deposition of the amyloid β-peptide (Aβ) in the brain is one of the hallmarks of Alzheimer’s disease (AD). It is not yet clear whether Aβ always leads to similar changes or whether it induces different features of neurodegeneration in relation to its intra- and/or extracellular localization or to its intracellular trafficking routes. To address this question, we have analyzed two transgenic mouse models: APP48 and APP23 mice. The APP48 mouse expresses Aβ_1-42_ with a signal sequence in neurons. These animals produce intracellular Aβ independent of amyloid precursor protein (APP) but do not develop extracellular Aβ plaques. The APP23 mouse overexpresses human APP with the Swedish mutation (KM670/671NL) in neurons and produces APP-derived extracellular Aβ plaques and intracellular Aβ aggregates.

**Results:**

Tracing of commissural neurons in layer III of the frontocentral cortex with the DiI tracer revealed no morphological signs of dendritic degeneration in APP48 mice compared to littermate controls. In contrast, the dendritic tree of highly ramified commissural frontocentral neurons was altered in 15-month-old APP23 mice. The density of asymmetric synapses in the frontocentral cortex was reduced in 3- and 15-month-old APP23 but not in 3- and 18-month-old APP48 mice. Frontocentral neurons of 18-month-old APP48 mice showed an increased proportion of altered mitochondria in the soma compared to wild type and APP23 mice. Aβ was often seen in the membrane of neuronal mitochondria in APP48 mice at the ultrastructural level.

**Conclusions:**

These results indicate that APP-independent intracellular Aβ accumulation in APP48 mice is not associated with dendritic and neuritic degeneration but with mitochondrial alterations whereas APP-derived extra- and intracellular Aβ pathology in APP23 mice is linked to dendrite degeneration and synapse loss independent of obvious mitochondrial alterations. Thus, Aβ aggregates in APP23 and APP48 mice induce neurodegeneration presumably by different mechanisms and APP-related production of Aβ may, thereby, play a role for the degeneration of neurites and synapses.

**Electronic supplementary material:**

The online version of this article (doi:10.1186/2051-5960-1-77) contains supplementary material, which is available to authorized users.

## Background

The deposition of amyloid Aβ-peptide (Aβ) in the human brain and the formation of neurofibrillary tangles (NFTs) are histopathological hallmarks of Alzheimer’s disease (AD) [1, 2]. Neuron loss, neuritic and synaptic degeneration are seen in addition to Aβ-plaque deposition and NFT formation and are assumed to represent the morphological correlative of cognitive decline [3–5].

Aβ is a proteolytic fragment derived from the amyloid precursor protein (APP) by β- and γ-secretase cleavage [6, 7]. Aβ is the major component of extracellular senile plaques in the AD brain [2] and it has been considered to play a central role in AD pathogenesis [8]. In addition to extracellular Aβ-deposition, intracellular Aβ occurs in nerve cells in the AD brain [9, 10] and in mouse models for AD [11–13]. The role of intracellular Aβ in neurodegeneration and the development of AD is discussed controversially. Mutant intracellular Aβ has been shown to induce hippocampal cell loss associated with endoplasmic reticulum stress and mitochondrial alterations in cell culture [14]. Memory impairment in APP-transgenic mice has been observed even after reduction of plaques. In these animals increased levels of intraneuronal Aβ were reported [15]. The new APP48 mouse model expresses a proenkephalin signal peptide (SPENK)-human wild type Aβ_42_ construct in neurons of the central nervous system (CNS), exhibits intracellular Aβ-aggregates in neurons in the absence of Aβ-plaques, and develops CA1 neuron loss and motor deficits [16]. The name APP48 mouse is misleading because Aβ is produced independent from APP in these mice but we used the name APP48 mouse here because this mouse model was already introduced to the scientific community with this name [16]. Although Aβ production in APP48 mice differs from APP-derived Aβ production and does not model AD, APP48 mice allow the analysis of intracellular Aβ toxicity independent of APP under artificial conditions. The APP23 mouse is an Aβ-plaque producing mouse model, which overexpresses human APP with the Swedish mutation (KM670/671NL) in CNS neurons. It exhibits dendrite degeneration, loss of CA1 neurons and of asymmetric synapses in the frontocentral cortex [17–19]. In this mouse model Aβ is generated by proteolytic processing of APP by β- and γ-secretases. It accumulates extracellularly in Aβ plaques and in intracellular aggregates [13, 20]. Together, these mouse models offer the possibility to compare the effect of Aβ placed into the endoplasmic reticulum and the Golgi apparatus in APP48 mice with Aβ cleaved from APP in a post-Golgi compartment [21, 22] in APP23 mice. Both mice have been demonstrated to express high amounts of the transgene mRNA in neurons of the neocortex and hippocampus in a similar distribution pattern [16, 20] providing comparable transgene expression levels in the respective neurons of these mouse models. Here, we investigated 1) any pathological abnormalities associated with APP-independent intracellular Aβ accumulation in APP48 mice and 2) whether APP and Aβ production by β- and γ-secretase is critical for neurodegeneration in APP23 mice rather than the mere presence of the Aβ peptide as in APP48 mice.

To address these objectives we studied dendritic morphology of frontocentral layer III pyramidal cells and synapse densities in the frontocentral cortex and the hippocampal sector CA1 of APP48 mice in comparison with APP23 and wild type mice. We determined the numbers of neurons in the frontocentral cortex as well as in the hippocampal sector CA1, and compared ultrastructural changes in neurons of APP23, APP48, and wild type mice.

## Methods

### Animals

APP48 mice were generated as described previously [16] and continuously back-crossed to C57BL/6. A murine Thy-1 expression cassette was used encoding the rat proenkephalin signal sequence followed by human wild type Aβ_1-42_ to drive neuron-specific expression of human wild type Aβ_1-42_. APP23 mice were generated as described previously [20] and continuously back-crossed to C57BL/6. The same murine Thy-1 cassette was used to drive neuron-specific expression of human APP751 with the Swedish double mutation 670/671 KM → NL. Heterozygous female APP23 mice (3 months, n = 12; 15 months, n = 17) and female and male APP48 mice (3 months, n = 12; 18 months, n = 17) were analyzed. As control, respective female and male wild type littermates of 3 (n = 14), 15 (n = 10), and 18 months (n = 10) were used.

Animals were treated in agreement with the German and Swiss laws for the use of laboratory animals.

### Tissue preparation and DiI tracing

For DiI tracing the brains of 3 and 18-months-old APP48, 3 and 15-month-old APP23, and of 3 and 15-18-month-old wild type mice were studied. Mice were anesthetized. Perfusion was performed transcardially with Tris-buffered saline (TBS) with heparin (pH 7.4) followed by the injection of 0.1 M PBS (pH 7.4) containing 2.6% paraformaldehyde (PFA), 0.8% iodoacetic acid, 0.8% sodium periodate and 0.1 M D-L lysine. The brains were removed in total and post-fixed in 2.6% phosphate-buffered PFA (pH 7.4) containing 0.8% iodoacetic acid, 0.8% sodium periodate and 0.1 M D-L lysine [23]. Three days later a single crystal (0.3 mm^3^) of the carbocyanine dye DiI (Molecular Probes, Eugene, OR, USA) was implanted into the left frontocentral cortex, 1 mm rostrally from the central sulcus, 2 mm laterally from the middle line and 1 mm deep in the cortex as reported earlier [18]. This dye allows precise Golgi-like tracing of neurons in post-mortem fixed tissue in a quality similar to in vivo tracing methods [18]. After incubation in 2.6% phosphate-buffered PFA for at least 3 months at 37°C, 100 μm thick coronal vibratome sections were cut. All sections of a given mouse brain were separately stored and continuously numbered. Sections were temporarily mounted in TBS for microscopic analysis.

### Microscopic and quantitative analysis

In layer III of the frontocentral cortex of the right hemisphere, contralateral to the implantation site of the tracer, the morphology of traced commissural neurons was examined. The traced neurons were assigned to different types according to their morphology [18] (Additional file 1). Then the number of traced commissural neurons of each type in wild type mice was counted and compared with that in APP23 and APP48 mice. For qualitative and quantitative analysis 10 consecutive sections (100-μm thickness each) representing a tissue block of 1 mm thickness were studied for each mouse. Analysis started at the anterior commissure setting the caudal limit of the investigated tissue block. For each coronal section, the medial boundary of the region investigated was set as the vertical line at the cingulum that separated the cingulate cortex from secondary motor cortex (M2). The horizontal boundary was set as the horizontal line separating the primary somatosensory cortex (S1) from the insular cortex.

For the qualitative analysis a laser scanning confocal microscope (Leica TCS NT, Leica, Bensheim, Germany) was used. Stacks of 2D images were superimposed digitally using the Image J Image Processing and Analysis software (NIH, Bethesda, MD, USA), and 3D data sets were generated for the visualization of neurons with their entire dendritic tree. For quantification, traced neurons in layer III were counted in the region of interest in 10 consecutive sections of the tissue block taken for qualitative and quantitative analysis using a fluorescence microscope (Leica DMLB, Leica). In so doing, we analyzed a cortex volume of 5–6 mm^3^ in each mouse. Mean and median values of the number of traced neurons were calculated and compared between wild type, APP23, and APP48 mice.

### Immunohistochemistry

Immunohistochemistry was performed for the visualization of Aβ pathology as well as dendritic morphology in APP48 and APP23 mice. After formic acid pretreatment free-floating sections were incubated in goat anti-mouse immunoglobulin (IgG) to block cross-reactions with intrinsic mouse IgG as previously described [24]. To detect Aβ-positive material the sections were stained with monoclonal antibodies specifically detecting the C-terminus of Aβ_42_ (MBC42 [25], 1/200). In APP23 mice anti-Aβ_17-24_ (4G8; 1/5000, Sigma-Aldrich, St. Louis, USA) was used to stain Aβ-deposits regardless of the Aβ_40_ or Aβ_42_ C-terminus. MBC40 ([25], 1/20) antibodies detecting exclusively the C-terminus of Aβ_40_ were used in APP23 mice as well. N-terminal-truncated and pyroglutamate modified Aβ_N3pE_ was detected with anti-Aβ_N3pE_ (polyclonal rabbit, 1/100, additional microwave pretreatment, IBL International GmbH, Hamburg, Germany [26]). Phosphorylation of serine 8 of Aβ was detected with antibodies against phosphorylated Aβ (pAβ; SA5434, 1/5; 1E4E11, 1/50, additional microwave pretreatment [27, 28]). The primary antibodies were detected with a biotinylated secondary antibody and the ABC complex (Vectastain, Vector laboratories (Burlingame, CA. USA)), and visualized with DAB [29]. Sections were mounted in Eukitt^©^ (Kindler, Freiburg, Germany). The immunostained sections were analyzed with a Leica DMLB fluorescence microscope (Leica, Bensheim, Germany). Positive and negative controls were performed.

### Protein extraction from brain tissue

Protein extraction was carried out from female APP23 (n = 4), APP48 (n = 4), and wild type littermates (n = 4) mouse brains, aged 9–11 months. Mice of this age were taken to demonstrate the differences in the biochemical distribution of Aβ in APP23 and APP48 mice.

Fresh frozen forebrain (0.4 g) was homogenized in 2 ml of 0.32 M sucrose dissolved in Tris-buffered saline (TBS) containing a protease and phosphatase inhibitor-cocktail (Complete and PhosphoSTOP, Roche, Mannheim, Germany) with Micropestle (Eppendorf, Hamburg, Germany) followed by sonication. The homogenate was centrifuged for 30 min at 14.000 × g at 4°C. The supernatant (S1) with the soluble and dispersible fraction not separated from one another was kept. The pellet (P1) containing the membrane-associated and the insoluble, plaque-associated fraction was resuspended in 2% SDS.

Ultracentrifugation of the supernatant S1 at 175.000 × g was used to separate the soluble, i.e. the supernatant after ultracentrifugation (S2), from the dispersible fraction, i.e. the resulting pellet (P2). The pellet P2 with the dispersible fraction was resuspended in TBS.

The SDS-resuspended pellet P1 was centrifuged at 14.000 × g. The supernatant (S3) was kept as membrane-associated SDS-soluble fraction. The pellet (P3) that remained was dissolved in 70% formic acid and dried in a vacuum centrifuge (Vacufuge, Eppendorf, Hamburg, Germany) and reconstituted in 100 μl of 2X LDS (lithium dodecyl sulfate) sample buffer (Invitrogen, Carlsbad, CA, USA) followed by heating at 70°C for 5 min. The resultant sample was considered as insoluble, plaque-associated fraction [30]. The total protein amounts of soluble, dispersible, and membrane-associated fractions were determined using BCA Protein Assay (Bio-Rad, Hercules, CA, USA).

### SDS-PAGE and western blot analysis

For SDS-PAGE, soluble (S2), dispersible (P2), membrane-associated (SDS-soluble; S3), and insoluble, plaque-associated (formic acid soluble; P3) fractions (50 μg total protein) were electrophoretically resolved in a precast NuPAGE 4-12% Bis-Tris gel system (Invitrogen). The protein load was controlled either by Ponceau S staining or β-actin (C4, 1/1000, Santa Cruz Biotechnology, Santa Cruz, CA, USA) immunoblotting.

Aβ was detected by western blotting with anti-Aβ_1-17_ (6E10, Covance, Dedham, USA, 1/1000). Blots were developed with an ECL detection system (Supersignal Pico Western system, ThermoScientific-Pierce, Waltham, MA, USA) and illuminated in ECL Hyperfilm (GE Healthcare, Buckinghamshire, UK).

### Aβ ELISA

For analysis of Aβ by ELISA, forebrain homogenates from APP23 and APP48 mice of each age group (2–3 months: n = 6 (APP23), 6 (APP48); 15–18 months: n = 7 (APP23), 8 (APP48)) were homogenized, centrifuged and loaded on sandwich ELISA plates for quantification of Aβ peptides (Aβ_42_: ELISA from Innogenetics, Ghent, Belgium) as previously described [18]. Standard curves were prepared with synthetic Aβ_1-42_ purchased from Bachem and diluted in extracts of non-transgenic mouse forebrain prepared in parallel as described above. Each sample was analyzed in duplicate.

### Stereology

Six APP23, six APP48, and six wild type mice at the ages of 3 and 15-18-months, respectively, were chosen randomly for stereology. One brain section of the frontocentral cortex already quantified for the number of DiI-traced neurons was selected by chance and stained with aldehyde fuchsin-Darrow red. Quantification of neurons was performed according to the principles of unbiased stereology [31]. The frontocentral cortex volume was defined as the volume of the subfields M2, M1, S1 starting at the level of the anterior commissure as described previously [18]. The CA1 volume was measured in serial 100 μm thick sections of the entire mouse brain at 5x magnification. Neurons were counted in three different, randomly chosen microscopic fields (40x objective magnification) of an aldehyde fuchsin - Darrow red stained section of the frontocentral cortex and CA1, respectively. For optical dissection, stacks of 10 images in 2 μm focus distance were generated for each microscopic field. Only those neurons having nuclei with dark and round nucleoli visible in the center of soma in one of the stack-images were considered for quantification using the ImageJ software (NIH, Bethesda, USA). The number of neurons in the frontocentral cortex and CA1 was calculated on the basis of the respective reference volumes and neuron densities.

### Electron microscopy, immunoelectron microscopy and semiquantitative assessment of synapse densities and mitochondrial alterations

100 μm thick vibratome sections of the frontocentral cortex and of the hippocampus from six wild type, six APP23, and six APP48 mice, aged 3 and 15–18 months respectively, were flat-embedded in Epon (Fluka, Germany). A second vibratome section from each animal and region was flat embedded in LR-White-Resin (Hard-grade Acrylic Resin; London Resin Company, Berkshire, UK). A part of the frontocentral cortex covering all six cortical layers was dissected under microscopic control and pasted on Epon blocks with a drop of Epon. Likewise, a part of the CA1 subfield of the hippocampus with adjacent stratum oriens and radiatum was cut and pasted on a second Epon block ultrathin sections were cut at 70 nm. Epon sections were block stained with uranyl acetate and lead citrate, and viewed with a Philips EM400T 120KV (Philips, Eindhoven, The Netherlands), a Zeiss EM10 (Zeiss, Oberkochen, Germany), or a JEM-1400 (JEOL, Tokyo, JP) electron microscope. LR-White sections were immunostained with anti-Aβ_42_ (MBC42) and anti-Aβ_1-17_ (6E10, Covance, Dedham, USA, 1 mg/ml) antibodies and visualized with anti-mouse secondary antibodies (Aurion Immuno Gold Reagents & Accessories, Wageningen, The Netherlands) labeled with 10 nm nanogold particles. Digital pictures were taken.

Digital pictures from Epon embedded sections were taken from 20 soma- and plaque-free neuropil areas located in layers II-VI at 4600-times magnification. The numbers of the symmetric and asymmetric synapses were counted and the length of the synapses was determined with the ImageJ software (NIH, Bethesda, USA). The synaptic density was determined separately for symmetric and asymmetric synapses according to DeFelipe et al. [32] (synaptic density = number of synapse-profiles in a given area / length of synaptic profiles). These semiquantitative data were used to compare the synaptic densities between the different mouse lines. Asymmetric and symmetric synapses were distinguished according to published criteria [33, 34].

Synaptic densities in the CA1 regions were measured in 10 randomly taken pictures of the stratum oriens and in 10 randomly taken pictures of the stratum radiatum at 4600-times magnification.

**Figure 1:**
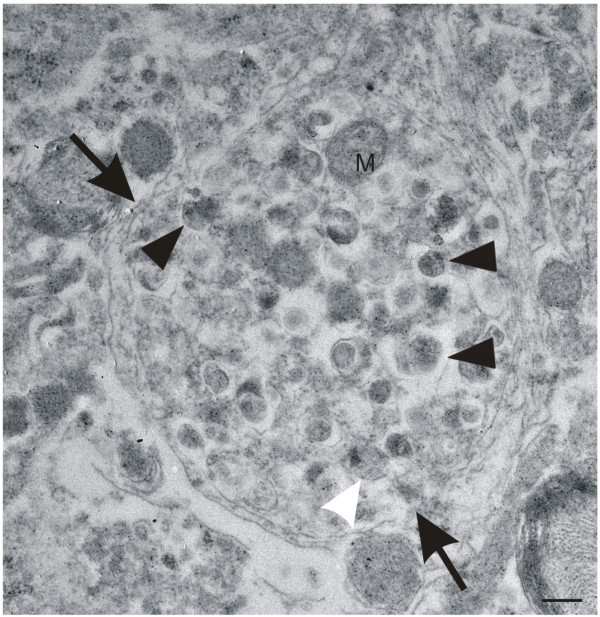
**Identification of dystrophic neurites.** Electron microscopy was used to identify dystrophic neurites (arrows) as previously described [19] and shown here in the frontocentral cortex of 15-month-old APP23 mice. Such neurites are characterized by neuritic swelling and contain vesicles with electron dense bodies (black arrowheads) probably representing autophagic vacuoles. Mitochondria in these neurites appear morphologically intact (M). Few multivesicular bodies are seen in these neurites as well (white arrowhead). The calibration bar corresponds to: 250 nm.

The frequency of dystrophic neurites was observed by counting the number of dystrophic neurites in the 20 pictures taken for the determination of the synapse densities. The criteria for the identification of dystrophic neurites at the ultrastructural level were: neurite profiles with a disorganized cytoplasm, occurrence of multilamellar structures in the absence of ultrastructurally intact cell organelles in the area of the lesion, and an enlarged size compared with neighboring neuritic profiles (Figure 1) [19]. Neurites of apoptotic or necrotic neurons, which are not enlarged and do not accumulate multilamellar bodies, were not considered as dystrophic neurites. The number of such dystrophic neurites was determined in six APP23, six APP48 and six wild type mice, aged 3 and 15–18 months respectively, and used as a semiquantitative score for structural neuritic alterations.

To clarify whether APP-independent production of intraneuronal Aβ or APP-derived extra- and intracellular Aβ accumulation are critical for mitochondrial changes in neurons we compared mitochondrial alterations in frontocentral and CA1 neurons of six APP23, six APP48 and six wild type mice, aged 3 and 15–18 months respectively, at the electron-microscopic level. For this purpose we measured the area profiles from neuritic and somatic neuronal mitochondria as well as from the respective somata and peripheral neurites in plaque-free areas. Mitochondria in peripheral dendrites and axons were analyzed in the 20 pictures from soma- and plaque-free areas of the frontocentral cortex and in 20 pictures of the stratum oriens and radiatum of the CA1 hippocampal subfield also used for the assessment of synapse densities. The mitochondria in neuronal somata were studied in 40 pictures from 10 different, randomly taken, layer II - layer VI neurons of the frontocentral cortex and in 40 pictures from 10 randomly taken CA1 neurons at 6000-times magnification (4 pictures per neuron) in each individual mouse. The area profiles from morphologically intact mitochondria and from those with an altered ultrastructure, i.e. degeneration of christae as described previously in prion disease [35], were separately obtained for somatic and peripheral neuritic mitochondria. Volume densities in percent of soma and neurite profiles were calculated according to the criteria for unbiased stereology [36] following the determinations provided in Table 1. Image processing and analysis software was used (ImageJ NIH, Bethesda, MD, USA) for this purpose. Assessments were performed without previous knowledge of the genotypes of the animals.

**Table 1:** **Determinations of the parameters employed for quantification of mitochondrial alterations in neurites and nerve cell somata**

Percentage of altered mitochondria in nerve cell somata =	
Percentage of altered mitochondria in neurites (axons and dendrites) =	
Mitochondrial volume density in the nerve cell somata =	
Mitochondrial volume density in neurites =	
Volume density of altered mitochondria in the nerve cell somata =	
Volume density of altered mitochondria in neurites =	

### Statistical analysis

SPSS 19.0 (SPSS, Chicago, IL, USA) software was used to calculate statistical tests. Non-parametric tests were used to compare wild type, APP23, and APP48 mice. p-values were corrected for multiple testing using the Bonferroni method. Parametric data were analyzed by ANOVA with subsequent Games-Howell post-hoc test to correct for multiple testing or using the Welch test. The results of the statistical analysis are summarized in Additional file 2.

## Results

### Different patterns of Aβ-pathology in APP23 and APP48 mice

**Figure 2:**
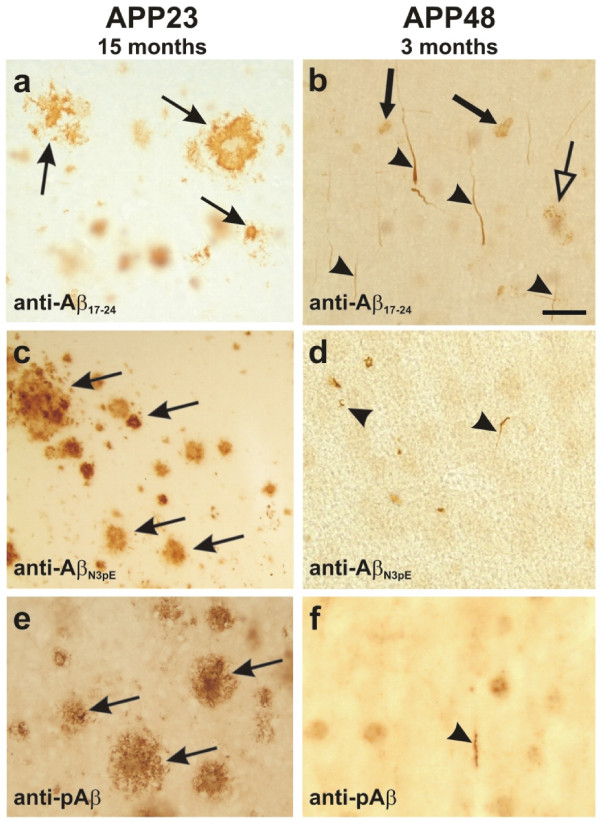
**Aβ-pathology in 15-month-old APP23 (a, c, e) and 3-month-old-APP48 mice (b, d, f). a**: The APP23 mouse showed a high number of extracellular Aβ plaques detectable with an antibody raised against Aβ_17-24_ (arrows). Intracellular Aβ was negligible. **b**: In the 3-month-old APP48 mouse no extracellular Aβ-pathology was apparent. These animals showed intraneuronal dendritic threads (arrowheads) and somatic granules (lucent arrow) as well as intramicroglial Aβ-grains (arrows) detectable with anti-Aβ_17-24_ as previously published [16]. **c**: Amyloid plaques in APP23 mice did also contain N-terminal truncated and pyroglutamate modified Aβ_N3pE_ (arrows). **d**: Aβ_N3pE_ was also found in even 3-month-old APP48 mice in some neuritic threads (arrowheads). **e**: phosphorylated Aβ (pAβ) was detected in amyloid plaques in 15-month-old APP23 mice. **f**: In 3-month-old APP48 mice only single threads showed labeling with anti-pAβ. Calibration bar in **b** corresponds to: **a, b** = 30 μm; **c** = 80 μm; **e** = 60 μm; **d, f** = 20 μm.

As previously published 15-month-old APP23 mice exhibited a high number of extracellular Aβ-plaques in the cerebral cortex (Figure 2a - indicated by arrows) as well as cerebral amyloid angiopathy as previously described in male and female animals [37]. Intracellular Aβ_42_ detectable with MBC42 was not abundant at the light microscopic level in this region neither in neurons nor in glial cells. MBC40-positive Aβ_40_ was observed in the perikarya of pyramidal neurons. At 3 months of age APP23 mice did not exhibit Aβ plaques or vascular Aβ deposits as previously described in male and female animals [37]. APP48 mice, on the other hand, did not show Aβ-plaques but intracellular accumulation of Aβ in dendritic threads, somatic granules in neurons, and in microglial Aβ-grains at 3 and 18 months of age (Figure 2b) [16]. This pathology was seen in male and female animals.

Modified Aβ such as Aβ_N3pE_ was detected in a large number of plaques in 15-month-old APP23 mice (Figure 2c). In APP48 mice only few dendritic threads exhibited Aβ_N3pE_ at 3 months of age (Figure 2d) whereas at 18 months of age a significant number of Aβ_N3pE_-positive inclusions was observed as shown previously [16]. Phosphorylated Aβ (pAβ) in APP23 mice was detected in plaques of 15 months old APP23 mice (Figure 2e). Intraneuronal pAβ in APP23 mice was apparent as previously reported in APP-PS1 transgenic animals [28]. Only single pAβ-positive threads were stained in 3-month-old APP48 mice (Figure 2f) whereas a few more pAβ-positive threads, grains and somatic granules were observed at 18 months of age.

**Figure 3:**
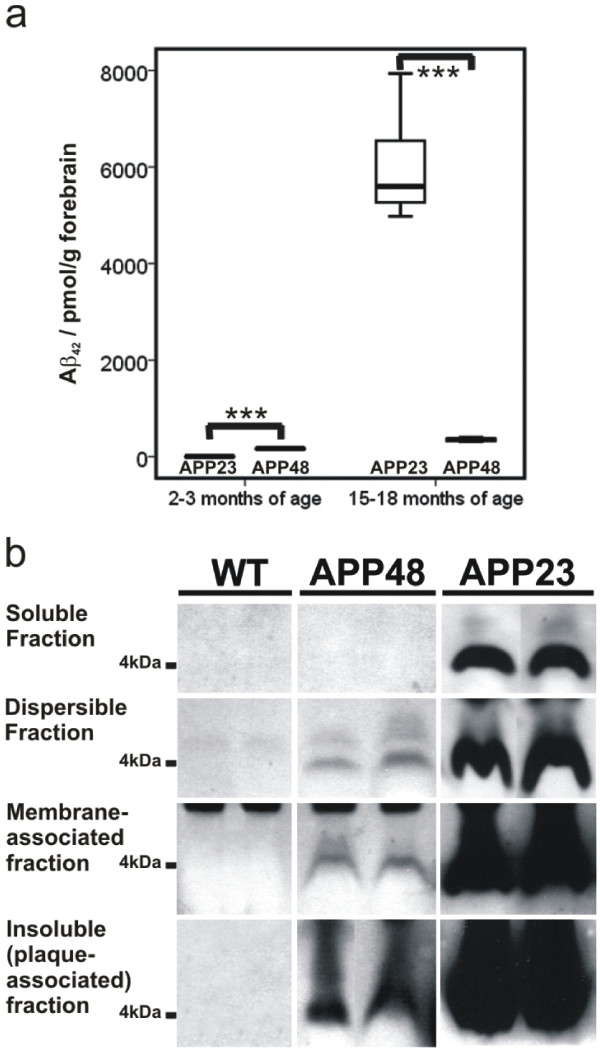
**Biochemical analysis of Aβ in APP23 and APP48 mice. a**: Total Aβ_42_ levels detected by ELISA in forebrain hemispheres of 2–3 and 15-18-month-old APP23 and APP48 mice. At 2–3 months APP23 mice exhibited low amounts of Aβ_42_ whereas APP48 mice displayed significantly more Aβ_42_ in the brain. At 15–18 months APP48 mice showed more Aβ than at 2–3 months of age but APP23 mice exhibited several times more Aβ in the forebrain. **b**: For demonstration of the types of Aβ aggregates in APP23 and APP48 mice brain homogenates of 9-11-month-old animals were analyzed by SDS-PAGE and western blotting after preparation of the soluble, dispersible, membrane-associated and insoluble (plaque-associated) fraction. Soluble Aβ as detected with antibodies raised against Aβ_1-17_ (6E10) was restricted to APP23 mice. Dispersible, membrane-associated, and insoluble (plaque-associated, formic acid soluble) Aβ aggregates were found in both transgenic mouse lines. The Aβ detected in the insoluble fraction of the forebrain homogenates of APP48 mice represents Aβ aggregates that require formic acid pretreatment before analysis similar to plaque-associated Aβ in APP23 mice. Since APP48 mice did not develop Aβ plaques this insoluble Aβ presumably represented intracellular fibrillar aggregates, such as dendritic threads. Wild type controls did not exhibit detectable amounts of Aβ in all four fractions. The original western blots are depicted in Additional file 3 (ELISA data from APP23 mice were previously published in a different context [18]). ***p < 0.001 Welch-test.

Biochemical analysis revealed that 3-month-old APP48 mice contained ~70 times more total Aβ_42_ detected by ELISA than APP23 mice whereas at 15–18 months of age APP23 mice contained Aβ_42_ in a concentration ~17 fold higher than in APP48 mice (Figure 3a, Additional file 2a).

To document the distribution of Aβ aggregates we analyzed brain homogenates of 9-11-month-old APP23 and APP48 mice for Aβ in the soluble (S2), dispersible (P2), membrane-associated (S3) and insoluble fraction (P3). APP23 mice exhibited soluble, dispersible, membrane-associated and insoluble, plaque-associated Aβ (Figure 3b) as reported previously in detail [19]. In contrast, APP48 mice only exhibited dispersible, membrane-associated and insoluble, aggregated Aβ whereas soluble Aβ was not detectable (Figure 3b).

### Degeneration of neurites and asymmetric synapses in APP23 but not in APP48 mice

**Figure 4:**
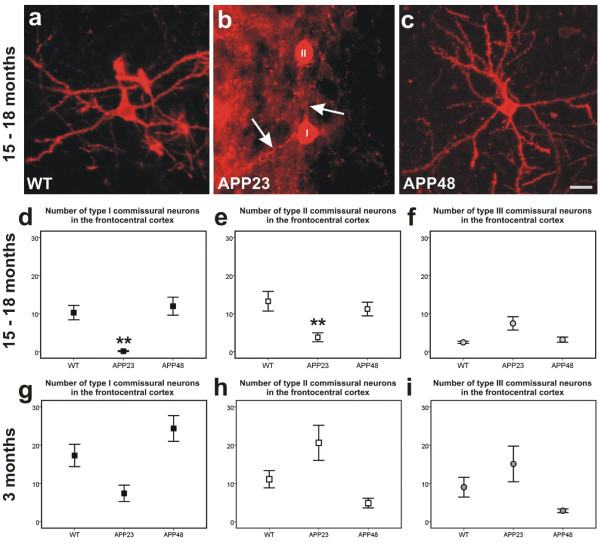
**Dendritic degeneration in frontocentral commissural neurons of APP23 and APP48 mice. a**: A type I neuron in an 18 month-old wild type animal exhibits a symmetric dendritic tree with prominent secondary and tertiary branches. **b**: In contrast, the dendritic tree of a representative type I commissural neuron (I) in a 15-month-old APP23 mouse is degenerated. Most basal dendrites were shrunken and had a reduced caliber (arrows). The degenerated dendrites showed some branches (arrows) that distinguished the degenerated type I neuron (I) from type II neurons without ramifications near the soma (II). **c**: Such a degeneration of the dendritic tree was not seen in APP48 mice. **d-f**: Numbers of DiI-traced type I, type II and type III commissural neurons in 15-18-month-old mice. **d**: APP23 mice at 15 months of age showed a decrease by more than 50% of the type I commissural neurons compared with 18-month-old wild type and APP48 mice. **e**: There was a significant reduction of type II commissural neurons in APP23 mice at 15 months of age compared with wild type littermates and APP48 mice at 18 months of age. **f**: Although APP23 mice had higher numbers of type III commissural neurons there was no significant difference from wild type littermates. **g-h**: No significant differences among the frequencies of DiI-traced type I, type II, and type III commissural neurons were observed at 3 months of age. ** p < 0.01 (Further statistical analysis: Additional file 2). Means and standard errors are depicted in **d-i**. (Quantitative data from APP23 mice and their respective wild type littermates were previously published in a different context [18]). Calibration bar in **c** corresponds to: **a-c** = 30 μm.

Using retrograde tracing with DiI three types of commissural neurons were subclassified as previously published (Additional file 1) [18] in APP23, APP48, and wild type mice. Type I and type II commissural neurons exhibited alterations in the dendritic tree as well as a decrease in number in 15-month-old APP23 mice compared to wild type littermates as previously reported [18] (Figure 4a-f, Additional file 2). Type III commissural neurons did not exhibit differences in their morphological appearance among APP48, APP23, and wild type littermates (Figure 4f). There were no significant differences in the numbers of type I, II, and III commissural neurons in 3- and 18-month-old APP48 mice and in 3-month-old APP23 mice compared to the respective wild type littermates (Figure 4c-i, Additional file 2b). Thus, 15-month-old APP23 mice exhibited dendritic degeneration of frontocentral commissural neurons whereas APP48 mice did not.

**Figure 5:**
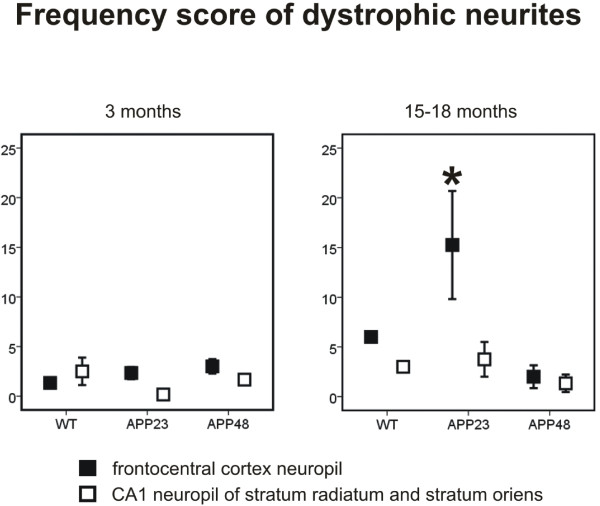
**Frequencies of dystrophic neurites in wild type, APP23, and APP48 mice.** The semiquantitatively assessed frequency of dystrophic neurites in the soma- and plaque-free frontocentral neuropil at the electron microscopic level was higher in 15-month-old APP23 mice than in wild type and APP48 mice of the same age group. In APP48 mice there was no increase in the frequency of dystrophic neurites in comparison to wild type mice. 3-month-old mice did not exhibit significant differences in the frequency of dystrophic neurites among the 3 genotypes nor were differences found in neuropil of the stratum radiatum and oriens of the CA1 region. (Data from APP23 mice and their respective wild type littermates were previously published in a different context [19]). *p < 0.05 (Further statistical analysis: Additional file 2). Means and standard errors are depicted.

To confirm neuritic degeneration we used transmission electron microscopy to compare the presence of dystrophic neurites among APP23, APP48 and wild type mice. The frequency of dystrophic neurites was higher in the frontocentral cortex of 15-month-old APP23 mice when compared to 15-18-month-old APP48 and wild type mice (Figure 5, Additional file 2c). There were no differences in the frequency of dystrophic neurites between APP48 and wild type mice or between 3-month-old animals of each genotype (Figure 5).

**Figure 6:**
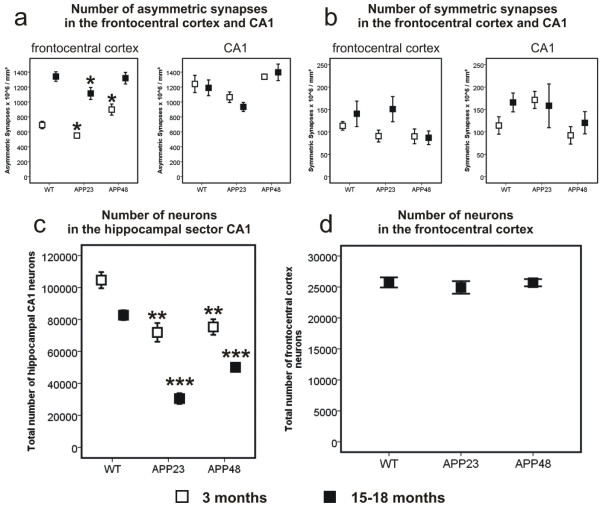
**Synapse densities in wild type, APP23 and APP48 mice. a**: Loss of asymmetric synapses in the frontocentral cortex of 3- and 15-month-old APP23 mice in comparison to wild type mice. 18-month-old APP48 and wild type mice did not differ significantly in the density of asymmetric synapses. At 3 months of age APP48 mice had even more asymmetric synapses than wild type animals. In CA1 there were also slightly less asymmetric synapses in APP23 mice than in wild type controls and APP48 mice. However, these differences were not significant. **b**: There were no significant differences in the number of symmetric synapses in the frontocentral cortex and in CA1 in 3- and 15-18-month-old animals. **c**: APP23 and APP48 mice of both age groups exhibited reduced numbers of CA1 neurons compared to wild type mice whereby CA1 neuron loss was most pronounced in APP23 mice. **d**: The number of neurons in the frontocentral cortex did not vary significantly among 15-18-month-old wild type, APP23, and APP48 mice. Therefore, younger animals were not studied for the number of neurons in the frontocentral cortex. *p < 0.05, **p < 0.01, ***p < 0.001 (Further statistical analysis: Additional file 2). Means and standard errors are depicted. (Some data were previously published in a different context [16, 19]).

Qualitative changes in synapse morphology other than the generation of dystrophic neurites in 15-month-old APP23 mice were not observed. Semiquantitative analysis of the densities of symmetric and asymmetric synapses showed a reduction of the density of asymmetric synapses in the frontocentral cortex of 3 and 15-month-old APP23 mice in comparison to WT mice. In the stratum radiatum and oriens of CA1 a similar trend was observed but did not reach significance. Such a reduction of asymmetric synapses was not observed in APP48 mice in comparison to wild type littermates. Moreover, 3-month-old APP48 mice exhibited more asymmetric synapses than wild type controls (Figure 6a, Additional file 2d). There were no significant differences in the numbers of symmetric synapses among APP23, APP48, and WT mice (Figure 6b, Additional file 2d).

The number of asymmetric synapses increased with age in the frontocentral neocortex of wild type, APP23, and APP48 mice (Figure 6a, Additional file 2d). Such an increase in the number of asymmetric synapses with age was not seen in the stratum radiatum and oriens of CA1 (Figure 6a, Additional file 2d). The number of symmetric synapses did not differ between 3 and 15-18-month-old mice of each genotype (Figure 6b, Additional file 2d).

**Figure 7:**
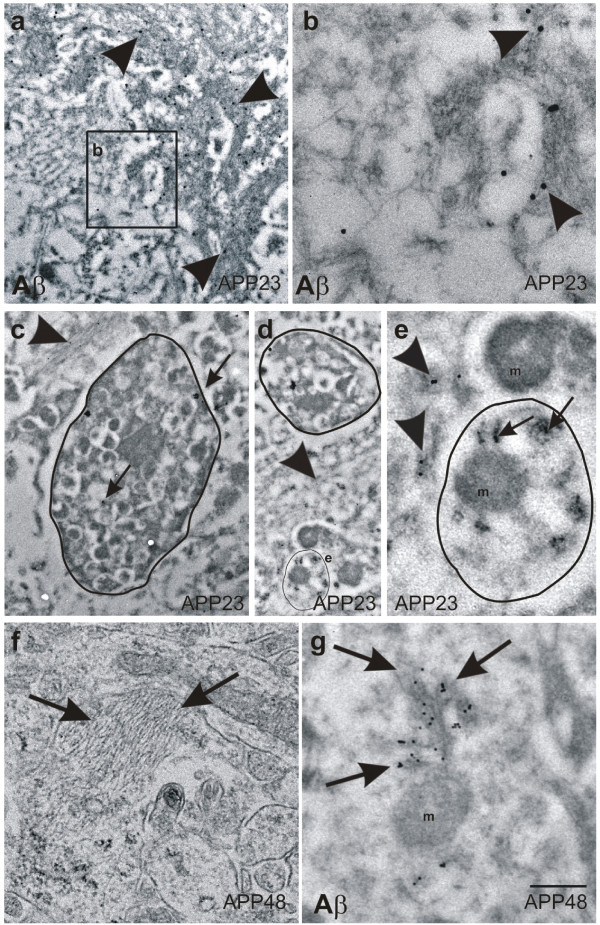
**Electron microscopy and immunogold labeling of Aβ in APP23 and APP48 mice. a**, **b**: Immunogold particles specifically labeled fibrillar Aβ (arrowheads) of a plaque in a 15-month-old APP23 mouse. At high magnification small amyloid fibrils were identified (arrowheads in **b**). They were located in the extracellular space. **c**, **d**: Dystrophic neurites (outlined structures) were associated with extracellular bundles of plaque-associated Aβ fibrils (arrowheads) in an 15-month-old APP23 mouse. Within the neurite, Aβ was localized in electron dense material near the surface as well as in the center of the neurite (arrows in **c**). **d**: A second dendrite without signs of dystrophy such as multilamellar bodies was also located near extracellular Aβ fibrils (outlined structure labeled with **e**). **e**: Higher magnification of this dendrite showed a dendrite cross section with an intact mitochondrium (m) and with condensed Aβ-positive material in the cytoplasm (arrows). Similar Aβ-positive material was found in the neighboring extracellular space (arrowhead). Both, intra- and extracellular Aβ aggregates did not exhibit fibrillar morphology. As such it is quite likely that these Aβ aggregates represent non-fibrillar oligomers and/or protofibrils occurring in the neighborhood of extracellular, plaque-associated Aβ fibrils. **f**: Fibrillar material (arrows) was observed in some dendrites of a 3-month-old APP48 mouse in an Epon-embedded, not immunostained section presumably representing the ultrastructural correlative for dendritic threads. **g**: Immunoelectron microscopy confirmed Aβ-positive material in fibrillar aggregates within dendritic threads labeled by gold particles (arrows) in APP48 mice. No Aβ was observed in the neighboring, non-altered mitochondrium (m). Calibration bar in **g** is valid for: **a** = 570 nm, **b** = 200 nm, **c** = 750 nm, **d** = 1000 nm, **e** = 275 nm, **f**, **g** = 350 nm.

Immunoelectron microscopy showed Aβ within dendrites of 15-18-month-old APP23 and APP48 mice. In 15-month-old APP23 mice extracellular Aβ plaques contained fibrillar Aβ that could easily be labeled with anti-Aβ_1-17_ (Figure 7a, b). Plaque-associated dystrophic neurites were seen in the middle of bundles of extracellular Aβ fibrils. No fibrillar Aβ was found within neurites of APP23 mice. However, intracellular Aβ was detected in a few of these dystrophic neurites near the membrane in electron dense spherical particles, which may represent non-fibrillar Aβ oligomers or protofibrils (Figure 7c-e). In APP48 mice fibrillar Aβ aggregates presumably representing the ultrastructural correlate of dendritic threads were found in the dendrites as seen morphologically in Epon-embedded tissue. These dendrites were not enlarged (Figure 7f). Immunoelectron microscopy with anti-Aβ_1-17_ indicated that the fibrillar structures identified in the Epon-embedded sections contain Aβ (Figure 7g) [16]. Organelles near dendritic threads in APP48 mice, thereby, did not differ from that elsewhere in APP48 mouse neurons as demonstrated for a mitochondrium in Figure 7g (m).

### Neuron loss in the CA1 subfield but not in the frontocentral cortex of APP48 and APP23 mice

The number of CA1 neurons was lower in 3- and 18-month-old APP48 mice compared to wild type animals indicating CA1 neuron loss in APP48 mice [16]. A decrease in the number of CA1 neurons was also observed in 3- and 15-month-old APP23 mice in comparison to wild type animals (Figure 6c, Additional file 2e) [19].

In contrast, the number of neurons in the frontocentral cortex of 15-18-month-old APP23 and APP48 mice was not significantly different from that in wild type mice (Figure 6d, Additional file 2e) [16]. Therefore, we did not analyze 3-month-old animals for the numbers of frontocentral neurons.

### Increased mitochondrial alterations in neuronal somata of APP48 but not of APP23 mice

**Figure 8:**
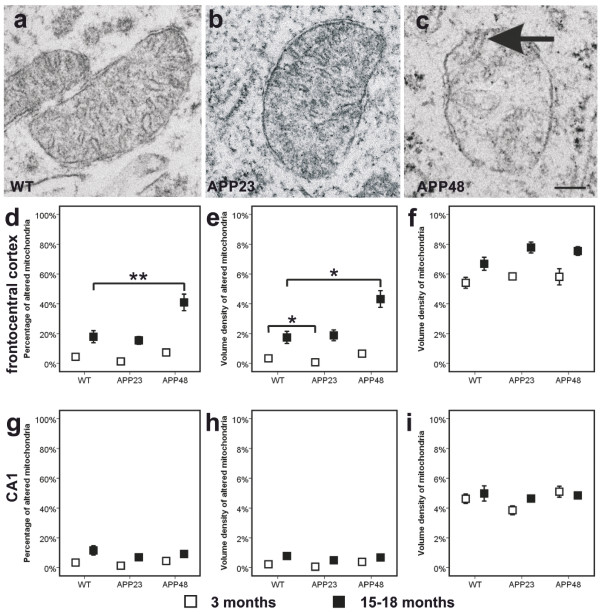
**Electron microscopy of mitochondria in wild type, APP23, and APP48 mice. a**-**b**: Electron microscopy showed predominantly non-altered mitochondria in nerve cell somata of wild type (WT) **(a)** and APP23 mice **(b)**. **c**: More altered mitochondria in the nerve cell somata were observed in frontocentral neurons of 18-month-old APP48 mice. Mitochondrial alteration was characterized by a loss of mitochondrial christae although the double membrane architecture and at least single christa structures (arrow) were preserved. **d-f**: The percentage of altered mitochondria in the nerve cell somata of 18-month-old APP48 mice was higher than in 15-18-month-old wild type and APP23 mice **(d)**. The volume density of altered mitochondria in the frontocentral nerve cell somata of 18-month-old APP48 mice was increased in comparison to 15-18-month-old wild type and APP23 mice **(e)**. At 3 months of age such differences in the presence of altered mitochondria were not observed. The total mitochondrial volume density, i.e. altered and non-altered mitochondria together, did not differ among the investigated mouse lines in both age groups **(f)**. **g-i**: In CA1 there were no obvious changes in the percentage and volume density of altered mitochondria in the nerve cell somata. *p < 0.05, **p < 0.01 (Further statistical analysis: Additional file 2). Means and standard errors are depicted in **d-f**. Calibration bar in **c** is valid for: **a-c** = 150 nm.

The analysis of mitochondrial alterations in neurites and the somata of frontocentral neurons showed differences between 15-18-month-old WT, APP23, and APP48 mice (Figure 8a-f). Increased percentages and volume densities of altered mitochondria exhibiting destruction and rarefaction of the christa membranes as depicted in Figure 8c were observed in somata of neurons from APP48 mice in contrast to predominantly ultrastructurally intact mitochondria in wild type and APP23 mice (Figure 8a, b, d, e, Additional file 2f,g). However, few altered mitochondria were also observed in wild type and APP23 mice. Such an increase in the percentage and volume density of altered mitochondria in APP48 mice was not observed in the hippocampal subfield CA1 and in the frontocentral neocortex of 3-month-old animals (Figure 8d, e, g, h, Additional file 2f, g). Neurites studied distant from nerve cell somata exhibited a small number of altered mitochondria in all mice but did not show differences in the percentage and volume densities of altered mitochondria among the three mouse lines at both ages and locations (Additional file 2i, j, Additional file 3a-c). The volume densities of somatic and neuritic mitochondria in general, i.e. unaltered and altered mitochondria together, did not significantly differ in APP23, APP48 and wild type mice (Figure 8f, i, Additional file 2h, k, Additional file 3c, f).

In the neuronal somata, the percentages and volume densities of altered mitochondria in frontocentral and CA1 neurons increased with age (Figure 8d, e, g, h, Additional file 2f, g). The volume densities of all, altered and healthy-looking mitochondria increased in APP23 and APP48 mice with age whereas in wild type animals such an increase was not observed (Figure 8f, i, Additional file 2h).

In neuritic processes, an increase or at least an increasing trend with age in the percentages and volume densities of altered mitochondria was observed in the frontocentral cortex of APP23, APP48 and wild type mice (Additional file 2k, Additional file 4a, b). In the stratum radiatum and oriens neurites of CA1, the percentages and volume densities of altered mitochondria in 15-month-old APP23 mice were lower than in wild type mice whereas no differences were observed between 3-month-old APP23 and wild type mice as well as between APP48 mice of any age and wild type mice (Additional file 2k, Additional file 4d, e). In neurites, the volume densities of all, healthy-looking and altered mitochondria did not increase with age (Additional file 2k, Additional file 4c, f).

**Figure 9:**
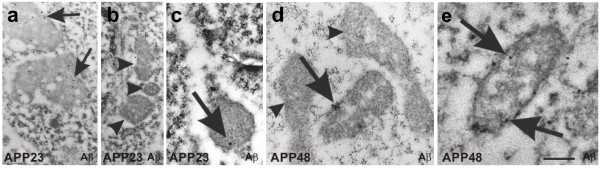
**Immunoelectron microscopy of mitochondria in wild type, APP23, and APP48 mice. a**: Immunoelectron microscopy revealed gold-labeled Aβ (arrows) within lipofuscin-like granules in the cytoplasm of frontocentral pyramidal neurons in 15-month-old APP23 mice. **b**. Most mitochondria in the somata of frontocentral pyramidal neurons in 15-month-old APP23 mice did not contain immunogold labeled Aβ-positive material (arrowheads). **c**: Only few neuronal mitochondria exhibited single gold particles in a 15-month-old APP23 mouse indicating anti-Aβ-positive material within these mitochondria (arrow). **d**: In a few mitochondria within frontocentral pyramidal nerve cell somata of 18-month-old APP48 mice we found immunogold labeled Aβ-positive material associated with the membranes of healthy-looking mitochondria (arrow). Neighboring non-altered mitochondria often did not contain Aβ (arrowheads). Aβ fibrils were not seen inside the mitochondria. **e**: Altered mitochondria within pyramidal neurons in the frontocentral cortex of a 18-month-old APP48 mouse also exhibited single gold particles in association with their membranes indicating the presence of Aβ (arrows) although Aβ fibrils were not observed inside the mitochondria. Due to the use of LR-white embedded tissue required for post-embedding immunoelectron microscopy **(a,b)** the tissue preservation was less good than in the Epon embedded sections used for the morphological analysis of the mitochondria (Figure 8 **a-c**). Calibration bar in **e** is valid for: **a, b** = 540 nm, **c** = 325 nm, **d** = 288 nm, **e** = 180 nm.

At the electron microscopic level Aβ was detected in lipofuscin granules or in association with other cytoplasmic material in neurons of APP23 (Figure 7c-e, Figure 9j, k) and APP48 mice as previously reported [16, 19, 38] with or without mitochondrial alterations. Even neurons of wild type mice without detectable Aβ contained single altered mitochondria. In 15-month-old APP23 mice Aβ was seen only in few mitochondria (Figure 9c) whereas most somatic and neuritic mitochondria did not contain Aβ-positive material (Figure 9b). In contrast, altered and non-altered mitochondria labeled for Aβ showing gold particles in association with mitochondrial membranes were often found in the nerve cell somata of 18-month-old APP48 mice (Figure 9d, e).

## Discussion

This study shows that APP-derived extra-and intracellular Aβ pathology in 15-month-old APP23 mice was associated with neuron loss, synapse loss and with neuritic alterations in non-apoptotic and non-necrotic neurons. On the other hand, APP-independent intraneuronal accumulation of Aβ in the absence of Aβ plaques in APP48 mice lead to a loss of neurons in the CA1 region [16] and to mitochondrial alterations but not to neuritic and synaptic degeneration in non-apoptotic and non-necrotic neurons. Both, extracellular plaques in APP23 mice and intraneuronal Aβ in APP48 mice also contained Aβ_N3pE_ and pAβ.

APP23 and APP48 mice differ considerably with respect to the mechanism of transgenic, human Aβ generation. Cleavage of Aβ from APP with the Swedish mutation as in APP23 mice occurs after APP internalization from the membrane in the early endosomal compartment [22, 39]. Once generated, Aβ is rapidly secreted into the extracellular space to a large extent. In APP48 mice, on the other hand, expression of Aβ_42_ with a cleaved signal sequence targets the released Aβ to the endoplasmic reticulum. Intracellular accumulation occurs in neurites and lysosomes whereas only very little Aβ_42_ is secreted into the extracellular space [16]. APP48 mice lack human APP and its metabolites except for Aβ_42_ whereas APP23 mice produce more Aβ_40_ than Aβ_42_ and, in addition to Aβ, N- and C-terminal fragments of APP. A transgenic mouse expressing only Aβ_40_ with a similar construct as used in APP48 mice for Aβ_42_ did not show Aβ aggregation and neuronal degeneration [16]. In double transgenic animals, expressing both Aβ_42_ and Aβ_40_ constructs, a similar pattern of pathological lesions was observed as in APP48 mice [16] indicating that Aβ_40_ has only a marginal effect in these animals.

Both, APP23 and APP48 mice exhibited intracellular Aβ in the lysosomal compartment and in mitochondria whereas early endosomal Aβ was not seen in APP48 mice. The distribution of the transgene mRNA in the neocortex and the hippocampus as well as the expression levels were quite similar in APP23 and APP48 mice [16, 20]. Accordingly, neocortical and hippocampal neurons did not vary much in the transgene expression levels in APP23 and APP48 mice. Both mouse models were used to determine the effects of Aβ under given artificial conditions. None of these two mouse models reflects human AD pathology because none showed significant neurofibrillary tangle pathology and both overexpressed a transgenic construct made to produce large amounts of Aβ [16, 20].

### Neurite and synapse degeneration in APP23 and APP48 mice

APP23 mice showed dendritic degeneration in DiI-traced commissural neurons, loss of asymmetric synapses and dystrophic neurites whereas none of these pathologies was observed in APP48 mice. In this context, it is important to note that fibrillar Aβ was present in dendrites of APP48 mice indicating that the mere presence of intracellular Aβ was not sufficient to induce this pathology. Accordingly, neuritic degeneration and loss of asymmetric synapses may either require high concentrations of extracellular Aβ_40/42_ and/or the presence of APP and its metabolites including APP-derived Aβ as in APP23 mice. The amount of total Aβ_42_ determined by ELISA did not explain neuritic degeneration because APP48 mice contained more Aβ_42_ in the brain than 3–5 months old APP23 mice (see also [18]) although 5-month-old APP23 mice did already exhibit dendrite pathology as previously reported [18]. Consistent with our observations, the prevalence of high levels of extracellular Aβ aggregates, e.g. soluble Aβ oligomers and protofibrils and Aβ plaques have been shown to be associated with altered synapse function [40–42]. Neuritic alterations were frequently seen near amyloid plaques [43, 44]. The electron microscopic detection of extracellular non-fibrillar and fibrillar Aβ-positive aggregates in association with dystrophic neurites in APP23 but not in APP48 mice further argues in favor of a critical role of extracellular Aβ in neuritic degeneration. Electrophysiological changes in response to extracellularly administered Aβ-aggregates were mainly reported for excitatory synapses [41, 42]. Since asymmetric synapses represent mainly excitatory (glutamatergic) synapses [34] the loss of these synapses likely represented the result of toxic interactions with Aβ aggregates. We have detected Aβ within few dystrophic neurites in APP23 mice indicating that we cannot exclude a contribution of intraneuronal Aβ to neuritic degeneration.

The Bri-Aβ_42_ mouse, that produces only extracellular Aβ_42_ derived from an ABri-Aβ_42_ construct did not show behavioral changes but Aβ plaques [45]. Bri-Aβ_40_ mice expressing an ABri-Aβ_40_ construct did not even develop plaques. These findings may argue against a contribution of extracellular Aβ to Aβ toxicity. However, it is not yet clear whether the ABri-derived Aβ_42_ aggregates contain similar amounts of Aβ_N3pE_ and pAβ as Aβ aggregates in APP23 or APP48 mice, whether APP expression or the presence of both, Aβ_42_ and Aβ_40_, is required for extracellular Aβ toxicity, or whether this mouse model did not produce enough Aβ_42_ to cause symptoms.

APP_E693Δ_ transgenic mice produce Aβ lacking glutamate-22 (E22Δ). This mouse model does not develop amyloid plaques but APP-derived intracellular Aβ aggregates and synapse loss [12]. Exogenous synthetic Aβ_E22Δ_ also lead to synaptic alterations in hippocampal slice culture experiments or after intraventricular injection [46, 47]. As such, neuritic changes and/or synapse pathology can be explained by APP-derived Aβ production regardless of the presence of Aβ plaques whereas such changes were not reported in APP48, Bri-Aβ_42_ and Bri-Aβ_40_ mice, which produce non-APP derived Aβ. A further argument for a role of APP in Aβ toxicity in APP23 mice is our recent finding of a coaggregation of Aβ with C-terminal APP fragments in dispersible Aβ aggregates [19] supporting the hypothesis that APP is a molecular target of Aβ toxicity [48].

### Mitochondrial alterations in APP23 and APP48 mice

APP-independent intraneuronal Aβ production and accumulation in 18-month-old APP48 mice was associated with more abundant structural mitochondrial alterations in somata of frontocentral nerve cells compared to APP23 and wild type mice. The increase of mitochondrial alterations in APP48 mice, however, was accompanied by the detection of Aβ in a moderate number of altered and non-altered neuronal somatic mitochondria whereas only few somatic mitochondria in APP23 mice exhibited Aβ and none in wild type animals. Several arguments suggest that the increase of mitochondrial alterations in APP48 mice was related to the presence of Aβ and may have functional consequences: 1) Aβ is capable of inducing apoptosis through the mitochondrial-caspase-3 pathway [49, 50], 2) mitochondrial Aβ levels are associated with the extent of mitochondrial dysfunction, oxidative stress and cognitive impairment in other AD mouse models and AD [51–55], and 3) histologically altered mitochondria showed a reduced number of christa membranes presumably providing a morphological correlate for an impairment of mitochondrial function; they were found most frequently in APP48 mice which also contained more mitochondrial Aβ than APP23 mice. For APP23 mice, however, we cannot rule out that trophic effects reported for APP expression [56] compensate Aβ toxicity to mitochondria.

It is noteworthy that only mitochondria in the nerve cell somata showed increased alterations in APP48 mice whereas mitochondria in distal dendrites and axons did not exhibit differences in volume density as well as in the percentage of alterations among APP23, APP48, and wild type mice. Thus, APP-independent, intraneuronal Aβ_42_ exhibited its major toxic effects on mitochondria close to its production site in APP48 mice, i.e. close to the endoplasmic reticulum. Biochemical assessment of respiratory chain complex I and complex IV, α-ketoglutarate dehydrogenase, and tricarboxylic acid cycle enzyme activity in APP48 and wild type mice did not show significant differences in forebrain homogenates (data not shown). However, the local effect in the soma is probably not sufficient to reduce the overall activities in a brain homogenate.

### Two types of Aβ-induced neurodegeneration in the frontocentral cortex: somatic type and neuritic type

**Figure 10:**
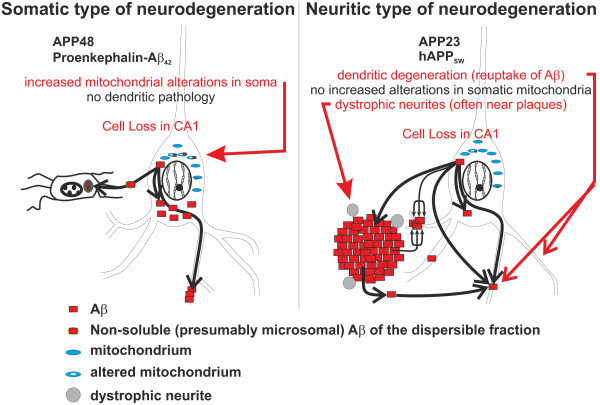
**Schematic representation of the somatic and neuritic type of Aβ-related neurodegeneration in frontocentral neurons of APP23 and APP48 mice.** In APP48 mice Aβ accumulates within neurons and in microglial cells as previously reported [16]. Extracellular Aβ is not detectable suggesting that APP-independently produced intracellular Aβ leads to functional impairment of neurons as indicated by motor deficits [16]. Mitochondrial alterations occur more frequently in the nerve cell somata of APP48 mice than in wild type and APP23 mice and are proposed to lead to apoptotic cell death as suggested previously [14] without preceding neuritic alteration. This type of somatic neurodegeneration in APP48 mice is different from that seen in APP23 mice, which contain less intracellular Aβ but significant amounts of extracellular Aβ aggregates including plaques. We propose that extra- and intracellular APP-derived Aβ causes synapse loss, dendrite degeneration and often plaque-associated, dystrophic neurites in APP23 mice indicative for a second neuritic type of neurodegeneration. Intracellular Aβ in APP23 mice may be produced within the nerve cell or may be taken up from the extracellular space [13, 57–59].

In the frontocentral cortex of APP23 mice neuritic degeneration and asymmetric synapse loss was found in the absence of neuron loss suggesting that both events indicate a neuritic type of nerve cell degeneration with neuritic degeneration preceding nerve cell death. APP48 mice, on the other hand, showed a different type of nerve cell damage characterized by morphologically altered mitochondria in the cell soma and thread-like Aβ aggregates in dendrites. Although the appearance of the dendrites and axons was, except for the Aβ threads, morphologically normal and no synapse loss was observed, mitochondrial changes in nerve cell somata represented early signs of a somatic type of neurodegeneration. Since mitochondrial alterations caused by Aβ have been demonstrated to induce apoptosis [49, 50] it is tempting to speculate that this type of somatic neurodegeneration with increased amounts of morphologically altered mitochondria finally results in apoptosis without the development of dystrophic neurites and dendrite degeneration before cell death. Hence, APP23 mice and APP48 mice develop two different types of nerve cell degeneration in the frontocentral cortex (Figure 10): 1) APP23 mice showed a neuritic type of neurodegeneration with early neuritic and synaptic degeneration but without increased numbers of altered mitochondria; 2) APP48 mice exhibited a somatic type of neurodegeneration with increased somatic mitochondrial degeneration but morphologically intact dendrites and axons.

APP48 and APP23 mice both showed neuron loss in the CA1 region as previously described [16, 17, 19]. It was not accompanied by a reduction in synaptic density suggesting replacement of lost synapses by surviving neurons. Except for Aβ plaques in APP23 mice and intracellular Aβ in both transgenic animals, significant reduction of synapse densities and mitochondrial alterations could not be identified in this brain region possibly because the more vulnerable CA1 neurons die faster than altered neurons in the frontocentral cortex and do not accumulate at early stages of nerve cell degeneration. Since 3-month-old animals have more CA1 neurons than 15-18-month-old mice (Figure 6c) and the decrease in number was higher than the age-related loss of CA1 neurons in wild type mice it appears likely that the age-related loss of CA1 neurons is enhanced by Aβ toxicity in APP23 and APP48 mice.

Although both types of Aβ-related neurodegeneration were observed in artificial mouse models that were made to produce large amounts of Aβ there are arguments for the hypothesis that both mechanisms of Aβ-related neurodegeneration are relevant in AD: 1) synapse loss and dystrophic neurites especially around neuritic plaques are well known features of AD pathology [3, 60, 61] that might be explained in part by the neuritic type of Aβ-related neurodegeneration and 2) mitochondrial alterations are well known in AD cases as well [54] presumably indicative for somatic type neurodegeneration in the presence of intracellular Aβ [10].

## Conclusions

Our data suggest two independent mechanisms by which Aβ causes neurodegeneration (Figure 10): a neuritic type and a somatic type. The neuritic type of neurodegeneration is characterized by a loss of asymmetric synapses, degeneration of dendrites, occurrence of dystrophic neurites and is associated with the occurrence of APP-derived extra- and intracellular Aβ aggregates in APP23 mice. The somatic type of neurodegeneration shows mitochondrial alterations in the neuronal soma but no changes in neurite morphology of non-necrotic and non-apoptotic cells. It is linked to intraneuronal accumulation of APP-independently produced Aβ and functional changes in APP48 mice [16]. Both mechanisms may finally lead to a loss of neurons as observed in the hippocampal sector CA1 in APP23 and APP48 mice [16, 17]. Although these mechanisms for Aβ-related neurodegeneration have been found under artificial conditions in Aβ producing mouse models it is tempting to speculate that similar mechanisms occur in AD. APP-related production of extra- and/or intracellular Aβ, thereby, appears to be critical for neuritic and synaptic degeneration. As such, for the development of therapeutic strategies aimed at protecting neurons from AD-related degeneration it appears important to consider both types of Aβ-related neurodegeneration.

## Electronic supplementary material

Additional file 1: **Types of commissural neurons in the frontocentral cortex as previously described** [18]**.** (DOC 26 KB)

Additional file 2: **Statistical analysis.** (DOC 108 KB)

Additional file 3: **Western blot analysis of soluble, dispersible, membrane-associated, and insoluble (plaque-associated) Aβ in wildtype, APP48 and APP23 mice.** Original blots related to Figure 3b. (PDF 5 MB)

Additional file 4: **Mitochondrial alterations in peripheral neurites.** **a**-**c**: Percentage of altered mitochondria (**a**), volume densities of altered mitochondria (**b**), and volume densities of all mitochondria (**c**) in peripheral neurites did not show significant changes among frontocentral neurons in wild type (WT), APP23 and APP48 mice. **d**-**f**: No significant differences in the percentage of altered mitochondria (**d**), volume densities of altered mitochondria (**e**), and volume densities of all mitochondria (**f**) in peripheral neurites of the CA1 sector of the Ammon’s horn were found between APP48 mice and wild type controls. 3-month-old APP23 mice had less morphologically altered mitochondria (**d**, **e**) than wild type controls. In 15-month-old animals the trend was still visible but did not reach significance (**d**, **e**). *p < 0.05 (Further statistical analysis: Additional file 2). (PDF 2 MB)
