# Supplementary material for: The type of Aβ-related neuronal degeneration differs between amyloid precursor protein (APP23) and amyloid β-peptide (APP48) transgenic mice
Source: Acta Neuropathol Commun. 2013 Nov 18;1(1):77. doi: 10.1186/2051-5960-1-77 (PMC4046770; doi:10.1186/2051-5960-1-77)
Supplement: Supplementary file 1 — Additional file 1: Types of commissural neurons in the frontocentral cortex as previously described [18]. (DOC 26 KB) [file 40478_2013_73_MOESM1_ESM.doc]

**Additional File. 1**: Types of commissural neurons in layer III of the frontocentral cortex identified by DiI-tracing [18]

| Type I commissural neurons | Pyramidal neurons with a highly ramified dendritic tree showing multiple secondary and tertiary branches |
| --- | --- |
| Type II commissural neurons | Pyramidal neurons with a dendritic tree that branches distant from the cell soma, secondary and tertiary branches are usually not observed in 100µm thick sections |
| Type III commissural neurons | Non-pyramidal commissural neurons |
