# Supplementary material for: The type of Aβ-related neuronal degeneration differs between amyloid precursor protein (APP23) and amyloid β-peptide (APP48) transgenic mice
Source: Acta Neuropathol Commun. 2013 Nov 18;1(1):77. doi: 10.1186/2051-5960-1-77 (PMC4046770; doi:10.1186/2051-5960-1-77)
Supplement: Supplementary file 2 — Additional file 2: Statistical analysis. (DOC 108 KB) [file 40478_2013_73_MOESM2_ESM.doc]

**Additional File 2**: Statistical analysis (significant differences are marked in red)

*a. Aβ1-42 levels detected by ELISA: Mean, standard deviation, Welch-test*

|  | **Mean Aβ1-42 (pmol/g)** | **p-value** |
| --- | --- | --- |
| APP23 – 2-3 months (n=4) | 2.25 +/- 0.36 | } <0.001 |
| APP48 – 2-3 months (n=6) | 165.00 +/- 6.48 |
| APP23 – 15 months (n=7) | 6019.74 +/- 1146.88 | } <0.001 |
| APP48 – 18 months (n=8) | 352.38 +/- 34.47 |

*b. DiI tracing of commissural neurons in the frontocentral cortex*

*DiI-tracing of commissural neurons: Mann-Whitney U-test (exact test – two sided)*

*Comparison between transgenic and wildtype mice*

| **3 months of age** | **Type I neurons** | **Type II neurons** | **Type III neurons** |
| --- | --- | --- | --- |
| APP23 mice vs wildtype littermates | p = 0.589 | p = 1.000 | p = 0.394 |
| APP48 mice vs wildtype littermates | p = 0.573 | p = 0.573 | p = 0.228 |

n = 6 (APP23, APP48); n = 5 (wildtype (APP23-littermates)); n = 8 (wildtype (APP48-littermates))

| **15-18 months of age** | **Type I neurons** | **Type II neurons** | **Type III neurons** |
| --- | --- | --- | --- |
| APP23 mice vs wildtype littermates | p = 0.001 | p = 0.004 | p = 0.093 |
| APP48 mice vs wildtype littermates | p = 0.422 | p = 0.235 | p = 0.134 |

n = 8 (APP23); n = 9 (wildtype (APP23-littermates)); n = 11 (wildtype (APP48-littermates)); n = 12 (APP48)

*DiI-tracing of commissural neurons: Mann-Whitney U-test (exact test – two sided)*

*Comparison between age groups*

| **3 months vs. 15-18 months** | **Type I neurons** | **Type II neurons** | **Type III neurons** | **n (3m; 15/18m)** |
| --- | --- | --- | --- | --- |
| Wildtype littermates APP23 | p = 0.012 | p = 0.689 | p < 0.001 * | 5; 9 |
| Wildtype littermates APP48 | p = 0.009 | p = 0.152 | p = 0.442 | 8; 11 |
| APP23 | p = 0.001 | p = 0.001 | p = 0.142 | 6; 8 |
| APP48 | p = 0.018 | p = 0.018 | p = 0.553 | 6; 12 |

*not considered different because not confirmed in APP48 wildtype littermates

*c. Frequency of dystropic neurites at the electron microscopic level*

*Comparison by genotype: ANOVA with Games-Howell post-hoc test*

|  | **frontocentral cortex** | | **CA1** | |
| --- | --- | --- | --- | --- |
|  | **3 months** | **15-18 months** | **3 months** | **15-18 months** |
| Wildtype vs. APP23 | 0.445 | 0.032 | 0.300 | 0.970 |
| Wildtype vs. APP48 | 0.198 | 0.939 | 0.829 | 0.228 |

n = 6 (each group)

*Comparison by age: Welch-test*

|  | **frontocentral cortex** | **CA1** |
| --- | --- | --- |
| Wildtype | 0.093 | 0.485 |
| APP23 | 0.004 | 0.015 |
| APP48 | 0.818 | 0.394 |

n = 6 (each group)

*d. Number of synapses: Mann-Whitney U-test (exact test – two sided)*

*Comparison by genotype*

|  | **frontocentral cortex** | | **CA1** | |
| --- | --- | --- | --- | --- |
| *Asymmetric synapses* | **3 months** | **15-18 months** | **3 months** | **15-18 months** |
| Wildtype vs. APP23 | 0.026 | 0.041 | 0.240 | 0.065 |
| Wildtype vs. APP48 | 0.041 | 1.000 | 0.818 | 0.240 |
| *Symmetric synapses* |  |  |  |  |
| Wildtype vs. APP23 | 0.240 | 0.589 | 0.093 | 0.394 |
| Wildtype vs. APP48 | 0.310 | 0.310 | 0.310 | 0.589 |

n = 6 (each group)

*Comparison by age*

| *Asymmetric synapses* | **frontocentral cortex** | **CA1** |
| --- | --- | --- |
| Wildtype | 0.002 | 0.699 |
| APP23 | 0.002 | 0.180 |
| APP48 | 0.009 | 0.937 |
| *Symmetric synapses* |  |  |
| wildtype | 1.000 | 0.093 |
| APP23 | 0.093 | 0.240 |
| APP48 | 0.937 | 0.699 |

n = 6 (each group)

*e. Number of hippocampal CA1 and of frontocentral neurons*

*Comparison by genotype: ANOVA with Games-Howell post-hoc test*

| *Frontocentral cortex* | **3 months** | **15-18 months** |
| --- | --- | --- |
| Wildtype vs. APP23 | n.a. | 0.812 |
| Wildtype vs. APP48 | n.a. | 0.999 |
| *CA1* |  |  |
| Wildtype vs. APP23 | 0.004 | <0.001 |
| Wildtype vs. APP48 | 0.004 | <0.001 |

n = 6 (15-18 months wildtype, APP23, APP48 and 3-month-old APP23, APP48); n = 7 (3-month-old wildtype)

*Comparison by age: Welch test*

|  | **Frontocentral cortex** | **CA1** |
| --- | --- | --- |
| Wildtype | n.a. | 0.005 |
| APP23 | n.a. | <0.001 |
| APP48 | n.a. | 0.002 |

n = 6 (15-18 months wildtype, APP23, APP48 and 3-month-old APP23, APP48); n = 7 (3-month-old wildtype)

*f. Percentage of altered somatic mitochondria: Mann-Whitney U-test (exact test – two sided)*

*Comparison by genotype*

| *Frontocentral cortex* | **3 months** | **15-18 months** |
| --- | --- | --- |
| Wildtype vs. APP23 | 0.065 | 0.937 |
| Wildtype vs. APP48 | 0.132 | 0.009 |
| *CA1* |  |  |
| Wildtype vs. APP23 | 0.394 | 0.394 |
| Wildtype vs. APP48 | 0.240 | 0.937 |

n = 6 (each group)

*comparison by age*

|  | **Frontocentral cortex** | **CA1** |
| --- | --- | --- |
| Wildtype | 0.015 | 0.026 |
| APP23 | 0.002 | 0.015 |
| APP48 | 0.002 | 0.004 |

n = 6 (each group)

*g. Volume density of altered somatic mitochondria: Mann-Whitney U-test (exact test – two sided)*

*Comparison by genotype*

| *Frontocentral cortex* | **3 months** | **15-18 months** |
| --- | --- | --- |
| Wildtype vs. APP23 | 0.026 | 0.818 |
| Wildtype vs. APP48 | 0.132 | 0.015 |
| *CA1* |  |  |
| Wildtype vs. APP23 | 0.240 | 0.310 |
| Wildtype vs. APP48 | 0.132 | 0.818 |

n = 6 (each group)

*Comparison by age*

|  | **Frontocentral cortex** | **CA1** |
| --- | --- | --- |
| Wildtype | 0.009 | 0.041 |
| APP23 | 0.002 | 0.002 |
| APP48 | 0.002 | 0.065 |

n = 6 (each group)

*h. Volume density of somatic mitochondria: Mann-Whitney U-test (exact test – two sided)*

*Comparison by genotype*

| *Frontocentral cortex* | **3 months** | **15-18 months** |
| --- | --- | --- |
| Wildtype vs. APP23 | 0.394 | 0.240 |
| Wildtype vs. APP48 | 0.699 | 0.180 |
| *CA1* |  |  |
| Wildtype vs. APP23 | 0.093 | 0.818 |
| Wildtype vs. APP48 | 0.394 | 0.589 |

n = 6 (each group)

*Comparison by age*

|  | **Frontocentral cortex** | **CA1** |
| --- | --- | --- |
| Wildtype | 0.394 | 0.818 |
| APP23 | 0.002 | 0.065 |
| APP48 | 0.026 | 0.485 |

n = 6 (each group)

*i. Percentage of altered neuritic mitochondria: Mann-Whitney U-test (exact test – two sided)*

*Comparison by genotype*

| *Frontocentral cortex* | **3 months** | **15-18 months** |
| --- | --- | --- |
| Wildtype vs. APP23 | 0.180 | 0.699 |
| Wildtype vs. APP48 | 0.132 | 0.132 |
| *CA1* |  |  |
| Wildtype vs. APP23 | 0.394 | 0.026 |
| Wildtype vs. APP48 | 0.310 | 0.394 |

n = 6 (each group)

*Comparison by age*

|  | **Frontocentral cortex** | **CA1** |
| --- | --- | --- |
| Wildtype | 0.041 | 0.394 |
| APP23 | 0.002 | 0.699 |
| APP48 | 0.180 | 0.485 |

n = 6 (each group)

*j. Volume density of altered neuritic mitochondria: Mann-Whitney U-test (exact test – two sided)*

*Comparison by genotype*

| *Frontocentral cortex* | **3 months** | **15-18 months** |
| --- | --- | --- |
| Wildtype vs. APP23 | 0.180 | 0.485 |
| Wildtype vs. APP48 | 0.394 | 0.132 |
| *CA1* |  |  |
| Wildtype vs. APP23 | 0.394 | 0.026 |
| Wildtype vs. APP48 | 0.394 | 0.394 |

n = 6 (each group)

*Comparison by age*

|  | **Frontocentral cortex** | **CA1** |
| --- | --- | --- |
| Wildtype | 0.065 | 0.394 |
| APP23 | 0.002 | 0.699 |
| APP48 | 0.065 | 0.065 |

n = 6 (each group)

*k. Volume density of neuritic mitochondria: Mann-Whitney U-test (exact test – two sided)*

*Comparison by genotype*

| *Frontocentral cortex* | **3 months** | **15-18 months** |
| --- | --- | --- |
| Wildtype vs. APP23 | 0.394 | 0.589 |
| Wildtype vs. APP48 | 0.699 | 0.818 |
| *CA1* |  |  |
| Wildtype vs. APP23 | 0.310 | 0.699 |
| Wildtype vs. APP48 | 0.394 | 0.093 |

n = 6 (each group)

*Comparison by age*

|  | **Frontocentral cortex** | **CA1** |
| --- | --- | --- |
| Wildtype | 0.394 | 0.310 |
| APP23 | 0.394 | 0.937 |
| APP48 | 0.240 | 0.065 |

n = 6 (each group)
