# Supplementary figures and images for: The type of Aβ-related neuronal degeneration differs between amyloid precursor protein (APP23) and amyloid β-peptide (APP48) transgenic mice
Source: Acta Neuropathol Commun. 2013 Nov 18;1(1):77. doi: 10.1186/2051-5960-1-77 (PMC4046770; doi:10.1186/2051-5960-1-77)

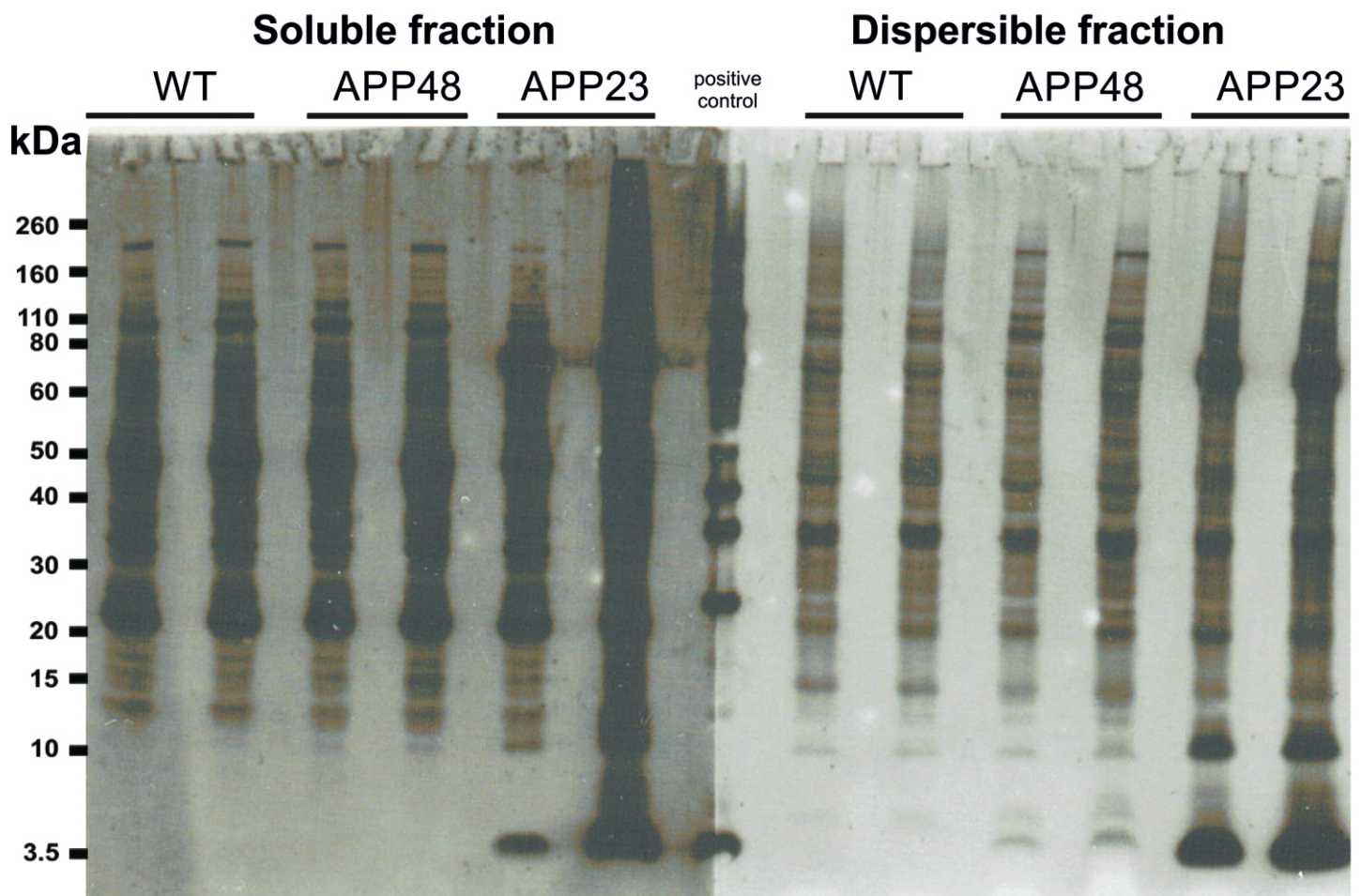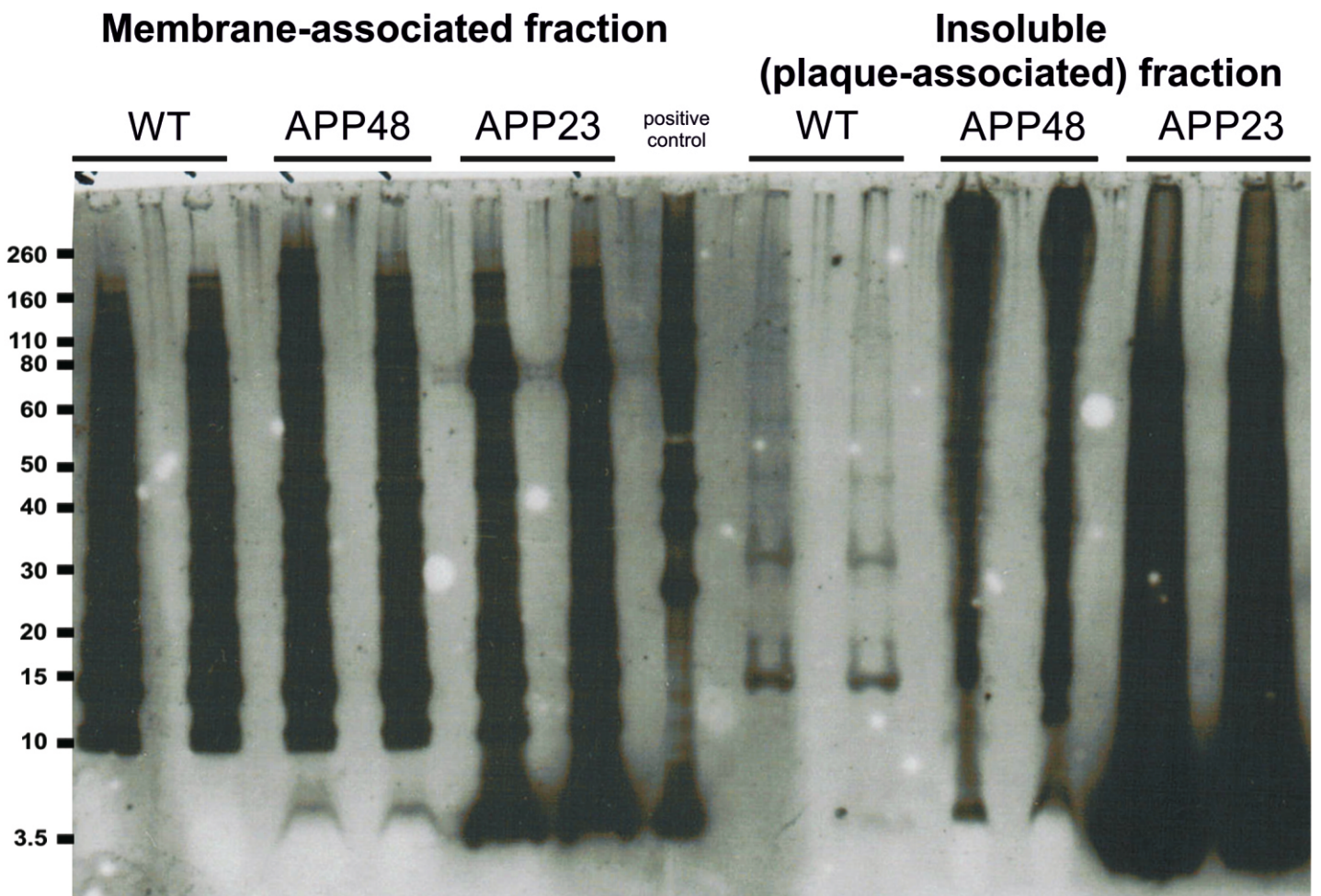

Supplement: Supplementary file 3 — Additional file 3: Western blot analysis of soluble, dispersible, membrane-associated, and insoluble (plaque-associated) Aβ in wildtype, APP48 and APP23 mice. Original blots related to Figure 3b. (PDF 5 MB) [file 40478_2013_73_MOESM3_ESM.pdf]

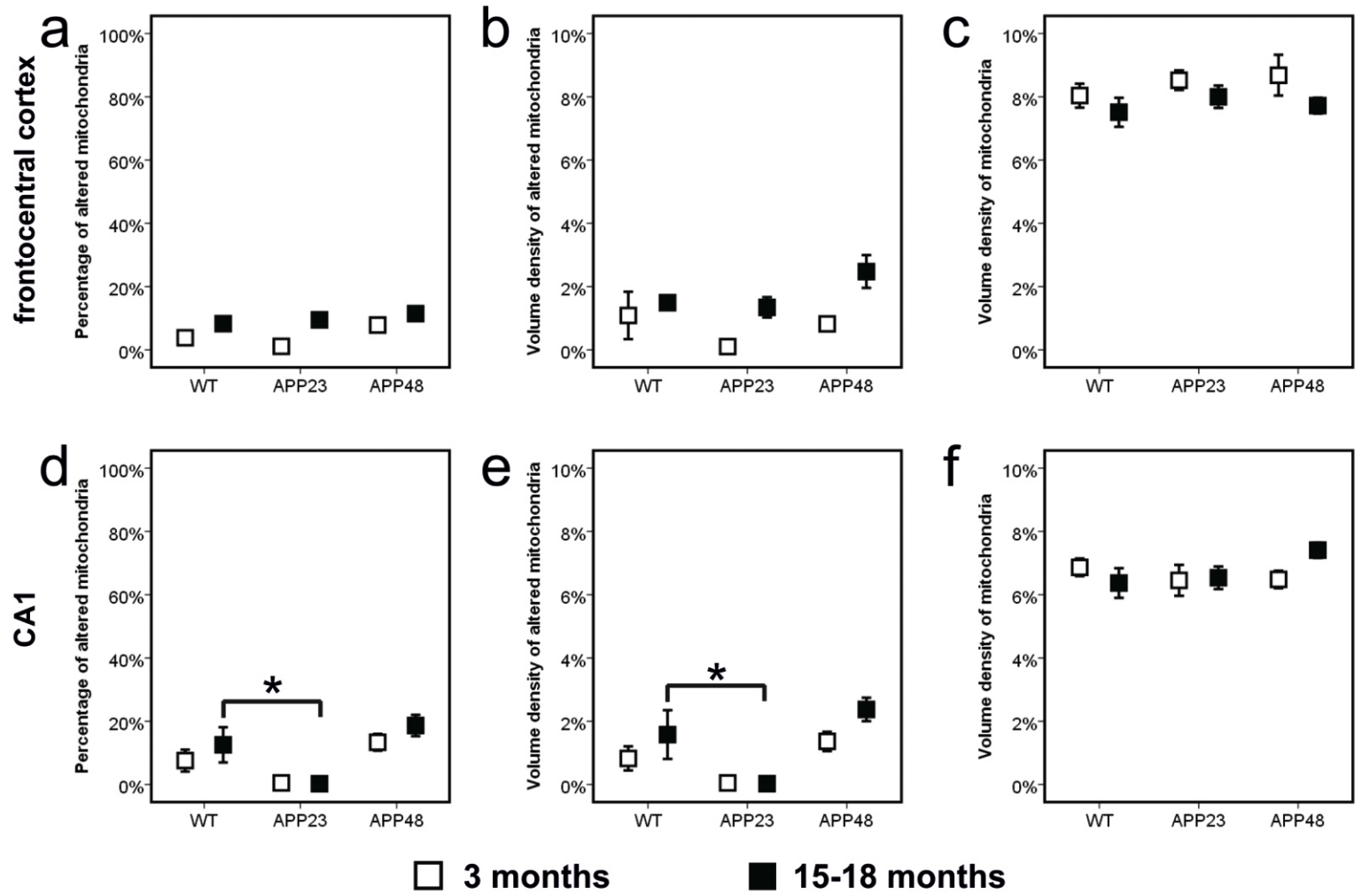

Supplement: Supplementary file 4 — Additional file 4: Mitochondrial alterations in peripheral neurites. a-c: Percentage of altered mitochondria (a), volume densities of altered mitochondria (b), and volume densities of all mitochondria (c) in peripheral neurites did not show significant changes among frontocentral neurons in wild type (WT), APP23 and APP48 mice. d-f: No significant differences in the percentage of altered mitochondria (d), volume densities of altered mitochondria (e), and volume densities of all mitochondria (f) in peripheral neurites of the CA1 sector of the Ammon’s horn were found between APP48 mice and wild type controls. 3-month-old APP23 mice had less morphologically altered mitochondria (d, e) than wild type controls. In 15-month-old animals the trend was still visible but did not reach significance (d, e). *p < 0.05 (Further statistical analysis: Additional file 2). (PDF 2 MB) [file 40478_2013_73_MOESM4_ESM.pdf]
